# Influenza virus immune imprinting dictates the clinical outcomes in ferrets challenged with highly pathogenic avian influenza virus H5N1

**DOI:** 10.3389/fvets.2023.1286758

**Published:** 2023-12-19

**Authors:** Ivette A. Nuñez, Hyesun Jang, Ying Huang, Alyson Kelvin, Ted M. Ross

**Affiliations:** ^1^Center for Vaccines and Immunology, University of Georgia, Athens, GA, United States; ^2^Department of Infectious Diseases, University of Georgia, Athens, GA, United States; ^3^Vaccine and Infectious Disease Organization (VIDO), University of Saskatchewan, Saskatoon, SK, Canada

**Keywords:** H5N1, pre-immunity, influenza imprinting, universal influenza vaccine, highly pathogenic avian H5N1 influenza A virus

## Abstract

Zoonotic transmission of H5N1 highly pathogenic avian influenza virus (HPAIV) into the human population is an increasing global threat. The recent 2022 HPAIV outbreak significantly highlighted this possibility, increasing concern in the general population. The clinical outcomes of H5N1 influenza virus exposure can be determined by an individual’s primary influenza virus infection (imprinting) or vaccination status. Immunological imprinting with Group 1 - (H1N1, H2N2, and H2N3) increases survival rates following H5N1 viral infection compared to Group 2 - (H3N2) imprinted individuals. Vaccination against H5N1 influenza viruses can offer protection to at-risk populations; however, stockpiled inactivated H5N1 influenza vaccines are not readily available to the public. We hypothesize that the immunological response to vaccination and subsequent clinical outcome following H5N1 influenza virus infection is correlated with the immunological imprinting status of an individual. To test this hypothesis, our lab established a ferret pre-immune model of disease. Naïve ferrets were intranasally inoculated with seasonal influenza viruses and allowed to recover for 84 days prior to H5N1 virus infection. Ferrets imprinted following H1N1 and H2N3 virus infections were completely protected against lethal H5N1 influenza virus challenge (100% survival), with few to no clinical symptoms. In comparison, H3N2 influenza virus-imprinted ferrets had severe clinical symptoms, delayed disease progression, and a sublethal phenotype (40% mortality). Consecutive infections with H1N1 influenza viruses followed by an H3N2 influenza virus infection did not abrogate the immune protection induced by the original H1N1 influenza virus infection. In addition, ferrets consecutively infected with H1N1 and H2N3 viruses had no clinical symptoms or weight loss. H3N2 pre-immune ferrets were vaccinated with a broadly reactive H5 HA-based or H1 NA-based vaccine (Hu-CO 2). These ferrets were protected against H5N1 influenza virus challenge, whereas ferrets vaccinated with the H1N1 wild-type CA/09 rHA vaccine had similar phenotypes as non-vaccinated H3N2-imprinted ferrets with 40% survival. Overall, Group 2 imprinted ferrets, which were vaccinated with heterologous Group 1 HA vaccines, had redirected immune responses to Group 1 influenza viral antigens and rescued a sublethal phenotype to complete protection.

## 1 Introduction

Primary influenza virus infection elicits a life-long imprint on the immune system, which further dictates serological and cellular immune responses upon re-infection (1). This imprinting, also known as “antigenic sin” (2) or “antigenic seniority” (3) is thought to dampen the serological response against subsequent infection with heterologous viral strains. Subsequent infection with influenza viruses recall antibody responses against shared antigens, even if the original antigen becomes a secondary or lesser component (4). The term original antigenic sin (OAS) has been correlated with negative effects, such that recall responses are targeted toward antigenic epitopes that have undergone antigenic drift, and recognition of the drift variant is lost (5). There is an ineffective cross-reactive immune response to new viral strains while maintaining protective antibody titers to the primary infection strain (6, 7). Some studies have shown that repeated vaccination results in a diminished antibody response to viral antigens (8, 9). However, these studies measured antibody protection through hemagglutinin inhibition assay (HAI) that only measures the antibody response against the hemagglutinin (HA) head to block sialic acid binding. Repeated vaccination can elicit antibody responses against HA-stem regions, which are not detectable via HAI (10). Following viral infection or seasonal vaccination, there is a pre-immune status that often narrows antibody cross-reactivity to influenza viruses. This is dependent on the age or date of birth of the individual (3, 11–13) but does not inhibit the immune response to unique influenza virus strains.

Although the induction of pre-immunity with seasonal influenza viruses has been previously investigated, the effects of pandemic H5N1 avian influenza virus (AIV) vaccination on a seasonal pre-immune background in ferrets are less well understood. There is an age-related response against H5N1 viral infection in humans with incidence and mortality due to H5N1 infection being highest in individuals born between the years 1957 and 2009 (14). Older adults with no record of prior exposure to AIV had heterotypic antibodies against H9N2 and H5N1 viruses (15). People immunized with seasonal influenza vaccines during the seasons between 2006 and 2011 had increased cross-protective antibodies (15). Out of the 174 participants, 25 had seroconverted levels of antibodies to the H5N1 (A/Vietnam/1194/2004) virus after seasonal influenza vaccination. However, out of those who had seroconverted only 1.1% had HAI antibody titers above 1:40 (15). It is speculated that because of the age of the individuals (74–79 mean age), they had been exposed to viruses similar to the Spanish influenza virus (H1N1 virus). This data is in agreement with the epidemiological study performed by Gostic et al. (14) that suggested individuals born after 1957 were most susceptible to H5N1 viral morbidity and were most likely to be imprinted with H3N2 viruses (14). This study found that primary infections of the seasonal influenza virus correlated with protection against the same influenza HA group, which is determined phylogenetically. Influenza virus HA proteins are antigenically divided into two groups: group 1, which contains viral subgroups H1, H5, and H2, and group 2, which contains H3, H7, and H9 (16). Individuals born before 1957, who were most likely imprinted with a group 1 influenza virus (H1N1) had lower case incidences of H5N1 (group 1) viral infection (14). Pre-immunity with group 1 or group 2 viruses leaves a long-lasting effect on the immunological response against exposure to heterologous viruses. This pre-immune status will ultimately influence pre-pandemic vaccination strategies and must be investigated in order to produce a successful H5N1 vaccine.

The development of a protective avian influenza vaccine has proven to be a difficult task. A pandemic avian influenza vaccine should be capable of inducing long-lasting memory response, neutralizing titers, cross-clade protection, and cellular and humoral responses in the occurrence of an outbreak. Human clinical trials with avian influenza vaccinations have shown varying results and complications. Avian influenza vaccines delivered as inactivated viruses are a safe approach to immunizing naïve populations but can also have negative setbacks. Inactivated vaccines take 6 months to produce and can often result in limited immunogenicity and elicit a poor cellular immune response (17). Inactivated vaccines are also often grown in eggs, which introduces a large amount of egg proteins and can sometimes have vaccine reactogenicity, especially in people who are younger than 23 years of age (18). Egg viral growth can also introduce glycosylation that is normally not present in the wild-type strain of the virus and can result in a vaccine that is not protective against circulating strains (19). The growth of HPAI viruses is also highly lethal to embryos and is often difficult to propagate safely in eggs (17). Most inactivated vaccines for H5N1 are poorly immunogenic and require at least two doses to elicit a long-lasting immune response (17). Unadjuvanted inactivated split virus and sub-unit H5N1 vaccines elicit neutralizing antibody titers in only 58% of individuals who are vaccinated (20). Antibody titers in these recipients decreased substantially 6 months after their second dose (20). The recipients were therefore offered a third dose to boost their antibody response, and protective titers were seen in 78 and 67% depending on the dose (21). Other studies have shown that the combination of inactivated influenza and adjuvant can induce a long-lasting response; however, the vaccine doses were very high, and neutralizing antibody titers were highest in children aged 6 months to 17 years of age (22, 23). Recombinant protein-based vaccines have shown promising results; rHA vaccines are well tolerated but do not easily elicit neutralizing antibodies after two doses (24). The current pre-pandemic vaccine that is stockpiled is a subvirion vaccine with recombinant H5N1 HA derived from the A/Vietnam/1203/2004 virus. Although this vaccine was well tolerated, it only induced neutralizing titers in 43% of the participants (20). Heterologous prime-boost regimens performed with this sub-unit vaccine, however, have been seen to induce cross-clade protection, specifically to clade 2.3.4.4 viruses (25, 26). However, there are still challenges to providing protection against highly variable and rapidly diverging strains of avian influenza viruses and a limited number of antigens that can be stockpiled.

In order to test vaccine efficacy and pre-immunity, an influenza model must be utilized. Fitch ferrets are commonly used as influenza experimental models. Although mice are most commonly used for influenza research, they lack clinical symptoms that follow influenza infection such as fever, sneezing, nasal discharge, and inflammation (27). The ferret is the only animal that displays clinical symptoms that are similar to those of humans. Ferrets were discovered as influenza models in 1933; they can be infected with influenza A viruses that do not require adaptation (28). Ferrets can also transmit influenza viruses to susceptible cage mates and carry the α-2,6 linked sialic acid receptors in their upper respiratory tract, which helps transmit the virus via airborne droplets (29, 30). Because of the extensive nature of housing ferrets, sample sizes for experimental purposes are often small (*n* = 4–5). The investigation of pandemic viruses such as H5N1 and H7N9 has increased the use of ferrets in influenza studies.

The effect of pre-immunity on pandemic vaccination has not yet been investigated. As previously mentioned, pre-immune status greatly determines an individual’s antibody production and repertoire and must be considered when developing a vaccine for pandemic preparedness. This study aimed to determine the protective response of group 1 vs. group 2 pre-immunity on H5N1 infection and the responses to vaccination depending on pre-immunity. Monovalent HA or NA recombinant proteins were used to vaccinate pre-immune ferrets prior to challenge. Specifically, the chimeric HA protein named Human COBRA 2 (computationally optimized broadly reactive antigen) was tested in H3N2 pre-immune ferrets in an attempt to offer complete protection. Our results show that ferrets that were convalescent for group 1 seasonal influenza virus maintained an antibody repertoire that was capable of neutralizing HPAI H5N1 virus challenge. Ferrets that were imprinted with a group 2 virus were not 100% protected against infection compared to the group 1 imprinted cohort. Sequential infection with the group 1 and group 2 viruses did not inhibit the imprinting effect and protection against H5N1 challenge.

## 2 Methods

### 2.1 Virus-like particle vaccine preparation

Mammalian 293 T HEK cells were transfected with each of three plasmids expressing the influenza neuraminidase A/Thailand/01/2004 (H5N1), the HIV p55 Gag sequences, and Human COBRA HA expressing plasmids on previously described mammalian expression vectors. After 72 h of incubation at 37°C, supernatants from transiently transfected cells were collected, centrifuged to remove cellular debris, and filtered through a 0.22-μm-pore-size membrane. Mammalian-derived VLPs were purified and sedimented by ultracentrifugation on a 20% glycerol cushion at 27,000 rpm for 4 h at 4°C. VLPs were resuspended in phosphate-buffered saline (PBS), and the total protein concentration was assessed by a conventional bicinchoninic acid assay. The hemagglutination activity of each preparation of VLPs was determined by adding an equal volume of Horse red blood cells (RBCs) to a V-bottom 96-well plate, followed by incubation with serially diluted volumes of VLPs for 60 min at room temperature.

### 2.2 Recombinant protein vaccine preparation

Soluble recombinant HA or NA was also produced for vaccination. The HA/NA gene cassettes expressing wild type or Human COBRA HA recombinant protein were cloned into mammalian DNA expression plasmid pcDNA 3.1/Zeo(+)vector (Thermo Fisher Scientific) and were synthesized by Genewiz (South Plainfield, NJ). The plasmid was transformed into a TOP10 bacterial cell line and was purified using Zympure maxi-prep. The HA1 fragment, which contained a KPNI site was removed from the plasmid and was moved into an acceptor vector containing the Hu-CO2 HA2 domain. The final gene of the HA protein contained an extracellular domain that was terminally fused with the trimeric domain of T4 fibritin, an AviTag sequence, and a hexahistidine affinity tag for purification (31). Each DNA plasmid containing either wild-type or COBRA antigens was transiently transfected into an Expi293F HEK suspension cell line (Thermo Fisher Scientific) and was allowed to incubate for 72 h at 37°C (5% CO2). Supernatants were collected and tested for protein expression through BCA and Western Blot (His tag antibody). The cells were then pelleted down and the supernatant was purified for protein collection. Soluble HA protein was purified via AKTA Pure System using HisTrap columns following the manufacturer’s protocol. Eluted fractions were pooled and purified, protein concentration was tested using an anti-HIS tag antibody (Biolegend, San Diego, CA, United States) using SDS-PAGE and Western blot (32).

### 2.3 Determination of hemagglutinin protein content in VLPS

Protein concentration was determined by MicroBCA™ Protein Assay Kit (Thermo Fisher Scientific; Pittsburgh, PA, United States). HA concentration was determined by western blot and densitometry. Purified VLPs were prepared in standard total protein amounts and were electrophoresed on 10% SDS-PAGE gel and transferred to a PVDF membrane. The blot was probed with an anti-HA clade 1 influenza A virus (Immune Technology Corporation; New York, NY, United States) monoclonal antibody. HA-antibody complexes were detected using a goat anti-mouse IgG conjugated to horse radish peroxidase (HRP) (Southern Biotech; Birmingham, AL, United States). HRP was detected by chemiluminescent substrate Clarity™ Western ECL substrate (Bio-Rad Laboratories; Hercules, CA, United States). The density of WT HA bands was used to calculate a standard curve and the density of the purified VLPs was interpolated using the results from the WT HA. Experiments were performed in duplicates. The density of bands was determined using myImageAnalysis™ Software (Thermo Fisher Scientific, Waltham, MA, United States).

### 2.4 Viruses

H1N1, H3N2, and H2N3 viruses were obtained through the Influenza Reagents Resource (IRR), BEI Resources, or the CDC or were provided by Sanofi-Pasteur. Viruses were passaged once under the same growth conditions as they were received in embryonated chicken eggs with the instructions provided by the WHO. Titers of virus lots were determined with both guinea pig and turkey erythrocytes and divided into aliquots for single-use applications. Viruses used to infect ferrets were as follows: H1N1: A/Singapore/6/1986 (A/Sing/86, 1×10^6^ pfu/mL), A/California/07/2009 (A/CA/09) and A/AA/Marton/1943 (A/Mar/43). H3N2: A/Panama/2007/1999 (A/Pan/99, 1×10^6^ pfu/mL), A/Port Chalmers/1/1973 (A/PC/73), A/Hong Kong/1/1968 (A/HK/68), A/Texas/50/2012 (A/TX/12). H2N3: A/Swine/Missouri/4296424/2006 (A/sw/MO/06). H5N1: A/Vietnam/1203/2004 (A/VN/04, 1×10^5^ pfu/mL).

### 2.5 Animal studies

Fitch ferrets (*Mustela putorius furo*, female and male, 6 to 12 months of age), were de-scented and purchased from Triple F Farms (Sayre, PA) or sent to us by our collaborator Dr. Alyson Kelvin from Dalhousie University. Ferrets were pair housed in stainless-steel cages (Shor-line Kansas City, KS) containing Sani-Chips laboratory animal bedding (P J. Murphy Forse Products, Montville, NJ). Ferrets were provided with Teklad Global Ferret Diet (Harlan Teklad, Madison WIS) and freshwater *ad libitum*. The University of Georgia Institutional Animal Care and Use Committee approved all experiments, which were conducted in accordance with the National Research Council’s *Guide for the Care and Use of Laboratory Animals*, The Animal Welfare Act, and the CDC/NIH *Biosafety in Microbiological and Biomedical Laboratories guide*. In order to determine negative immunological status, ferrets were bled 2 weeks prior to study initiation, and RDE-treated sera were tested for hemagglutinin inhibition assay-detectable antibodies against a panel of seasonal influenza viruses (H1N1, H3N2, H5N1, H5N2, H5N6, H5N8) and influenza B viruses (data not shown). Ferrets (*n* = 6–8) were intranasally inoculated with an H1N1 seasonal isolate or an H3N2 isolate. Animals were monitored for weight loss, loss of activity, nasal discharge, sneezing, and diarrhea and were allowed to recover for a predetermined time of 84 days. The recovery date was empirically determined prior to this study (unpublished) and since has been used in multiple models of pre-immunity (9–12). Ferrets were tested on days 30, 45, 60, and 84 post-infection for infection and vaccine immunogenicity. Ferrets that recovered withing 30-45 days did not experience viral infection or elicit antibodies against vaccine-specific antigens when immunized. Serum HAI titers tested 60 days post-infection showed significantly reduced reactivity, and by day 84, pre-immunized ferrets elicited vaccine-antigen-specific antibodies and experienced viral infection post-inoculation, which was determined by weight loss, fevers, and clinical symptoms. On day 84, ferrets were vaccinated with either VLP, rHA antigens, or inactivated seasonal influenza vaccine intramuscularly. Two weeks following vaccination, ferrets were bled to assess the serological antibody response against homologous and heterologous avian influenza strains. If antibody titers are not elicited after one vaccination, ferrets may have to be boosted for a second time. Vaccines consisted of 15 μg or rHA formulated with Addavax™ adjuvant at a 1:1 ratio. Four weeks after final vaccination, ferrets were challenged intranasally with 1 × 10^4^ or 1×10^5^ plaque-forming units (PFU) of the highly pathogenic H5N1 virus A/VN/2004 (Clade 1) in a volume of 0.5 mL in each nostril for a total infection volume of 1 mL. Ferrets were monitored daily for weight loss, disease signs, and death for 14 days after infection. Individual body weights, sickness scores, and death were recorded for each group on each day after inoculation. Sickness scores were determined by evaluating activity (0 = normal, 1 = alert and active with stimulation, 2 = alert but not active after stimulation, 3 = not alert or active after stimulation), nasal discharge (0 = absent, 1 = present), sneezing (0 = absent, 1 = present), decreased food intake (0 = absent, 1 = present), diarrhea (0 = absent, 1 = present), dyspnea (0 = absent, 1 = present), and neurological symptoms (0 = absent, 3 = present). Nasal washes were conducted on day 3 after inoculation. Washes were collected and stored at −80°C until use. The experimental endpoint was defined as >25% weight loss, development of neurological symptoms, or an activity score of 3 (not active or alert after stimulation). All H5N1 influenza virus studies were performed under high-containment biosafety level 3 enhanced conditions (BSL3+). All procedures were in accordance with the NRC Guide for the Care and Use of Laboratory Animals, the Animal Welfare 3,046 B.M. Giles, T.M. Ross / Vaccine 29 (2011) 3043–3,054 Act, and the CDC/NIH Biosafety in Microbiological and Biomedical Laboratories.

### 2.6 Elisa

Immulon 4HBX plates (Thermo Fisher) were coated overnight at 4°C with cH6/1 in carbonate buffer (pH 9.4) at 0.5 μg/mL containing 5 μg/mL fraction V bovine serum albumin (BSA [Equitech-Bio, Kerrville, TX]; 50 μL/well) in a humidified chamber. The plates were then blocked with 200 μL/well of ELISA blocking buffer (PBS containing 0.2% BSA plus 0.1% bovine gelatin and 0.05% Tween 20) for 1.5 h at 37°C. Serum samples were serially diluted in blocking buffer, and the plates were incubated overnight at 4°C. The plates were washed three times with PBS-Tween (PBS-T, 0.05%). Then, 100 μL/well of biotinylated goat anti-ferret IgG H&L HRP (Cambridge, MA) diluted at 1:10000 in blocking buffer was added, and the plates were incubated for 1 h at 37°C. The plates were washed four times with PBS-T. Then, 100 μL of ABTS [2,2-Azinobis-(3-ethylbenzthiazolinesulfonic acid); AMRESCO, Solon, OH] substrate with 0.16% H_2_O_2_ was added, and the plates were incubated at 37°C for 25 min. Colorimetric conversion was terminated by the addition of 5% SDS (50 μL/well), and the optical density was measured at 414 nm using a spectrophotometer (BioTek, Winooski, VT). After subtraction of the background, endpoint titers were determined as the reciprocal dilution of the last well, which had an OD414 above the mean OD414 plus three times the standard deviations of naïve animal sera.

### 2.7 Plaque assays

Plaque assays were performed in a high-level biosafety containment facility. Lung samples and nasal wash samples taken on day 3 post-infection were snap frozen and kept at −80°C until processing. Lungs were homogenized using a plunger and 0.2 μm strainer. Madin-Darby canine kidney (MDCK) cells were seeded 24 h prior to use at (5×10^5^) in each well of a six-well plate. The nasal wash and lung homogenate samples were diluted (final dilution factors of 100 to 10^6^) and overlaid on the cells in 200 μL of Dulbecco’s modified Eagle medium supplemented with penicillin–streptomycin, followed by incubation for 1 h in 37°C with 5% CO2. Samples were removed, the cells were washed twice, and the medium was replaced with 2 mL of L15 medium plus 0.8% agarose (Cambrex, East Rutherford, NJ), followed by further incubation for 72 h at 37°C with 5% CO2. The agarose was removed and discarded. The cells were fixed with 10% buffered formalin for 10 min and then stained with 1% crystal violet for 5 min. The plates were then thoroughly washed in distilled water to remove excess crystal violet before being air-dried; the number of plaques was then counted, and the number of PFU per milliliter was calculated.

### 2.8 H&E staining

To assess the viral replication and pathological effect of infection, mice (*n* = 3) were euthanized 3 days post-infection. The right lung lobes were taken for viral plaques and the incision was clamped with a hemostat; a 22-gauge needle was then used to puncture the apex of the heart and sterile PBS was perfused throughout the mouse for 2–3 min. After the blood was efficiently removed from the lungs, 10% formalin was then perfused to fix the left lobes. Lungs were removed and placed into formalin for 1 week prior to paraffin embedding. Mouse lungs were embedded into paraffin and were cut using a Lecia microtome. Transverse 5 μm sections were placed onto Apex superior adhesive glass slides (Leica biosystem Inc., IL, United States), which were coated for a positive charge, and were processed for H&E staining. Sections were deparaffinized in Xylene and hydrated using different concentrations of ethanol (100, 95, 80, and 75%) for 2 min each. Deparaffinized and hydrated lung sections were stained with Hematoxylin (MilliporeSigma, MA, United States) for 8 min at RT, differentiated in 1% acid alcohol for 10 s, and then counterstained with Eosin (Millipore sigma, MA, United States) for 30s; slides were dehydrated with 95 and 100% ethanol, cleared by Xylene, and mounted using Permount^®^ mounting media (Thermo Fisher Scientific, MA, United States).

## 3 Results

### 3.1 Influenza group imprinting determines disease and survival in a pre-immune ferret model

**Figure 1:**
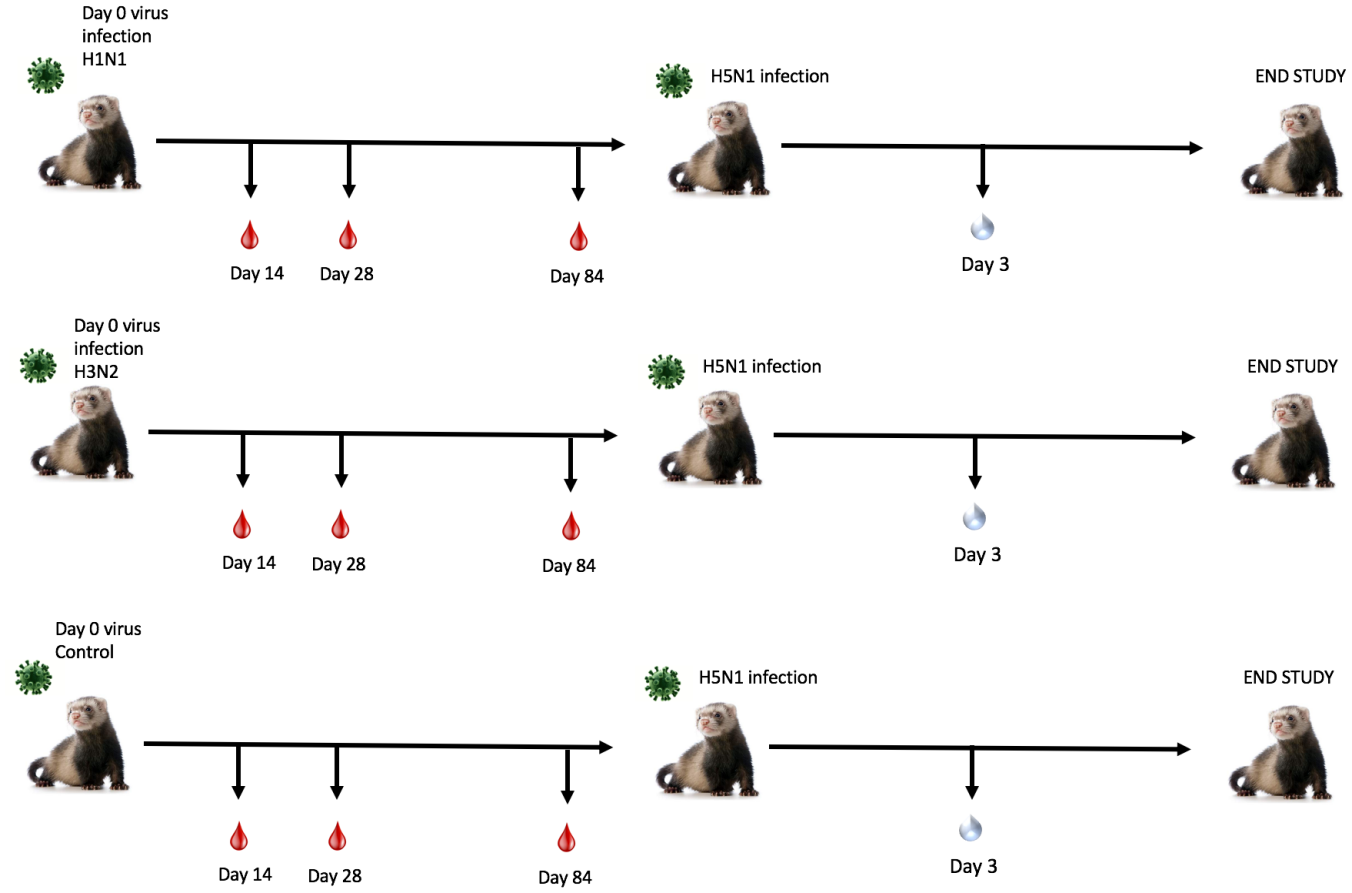
Schematic representation of pre-immune ferret study. Naïve fitch ferrets (*Mustela putorius furo*, female) 6–8 months of age were intranasally infected with a Group 1 influenza virus (H1N1 A/Sing/86), Group 2 influenza virus (H3N2 A/Pan/99), or PBS (control) and were monitored for 14 days following infection. Sera was collected to test for antibody responses on days 14, 28, and 84 post-preimmunization. Ferrets were allowed to recover for 84 days post-challenge. Ferrets were transported into a high-level biocontainment facility for H5N1 challenge with VN/04 (1×105 pfu/ml) and were monitored daily for weight loss and clinical symptoms. On day 3 post-challenge, ferrets were briefly anesthetized, and nasal wash specimens were taken to test for viral titers. The study was terminated 14 days post-challenge.

To determine the effect of seasonal influenza A virus imprinting on pandemic H5N1 infection, naïve female ferrets (*n* = 6) were intranasally inoculated (imprinted) with either an H1N1 influenza virus (A/Sing/86), an H3N2 influenza virus (A/Pan/99), or PBS as a control (Figure 1). Following seasonal influenza virus inoculation, ferrets were allowed to convalesce for 84 days and were then transported to a high-level animal biosafety facility (ABSL-3).

**Figure 2:**
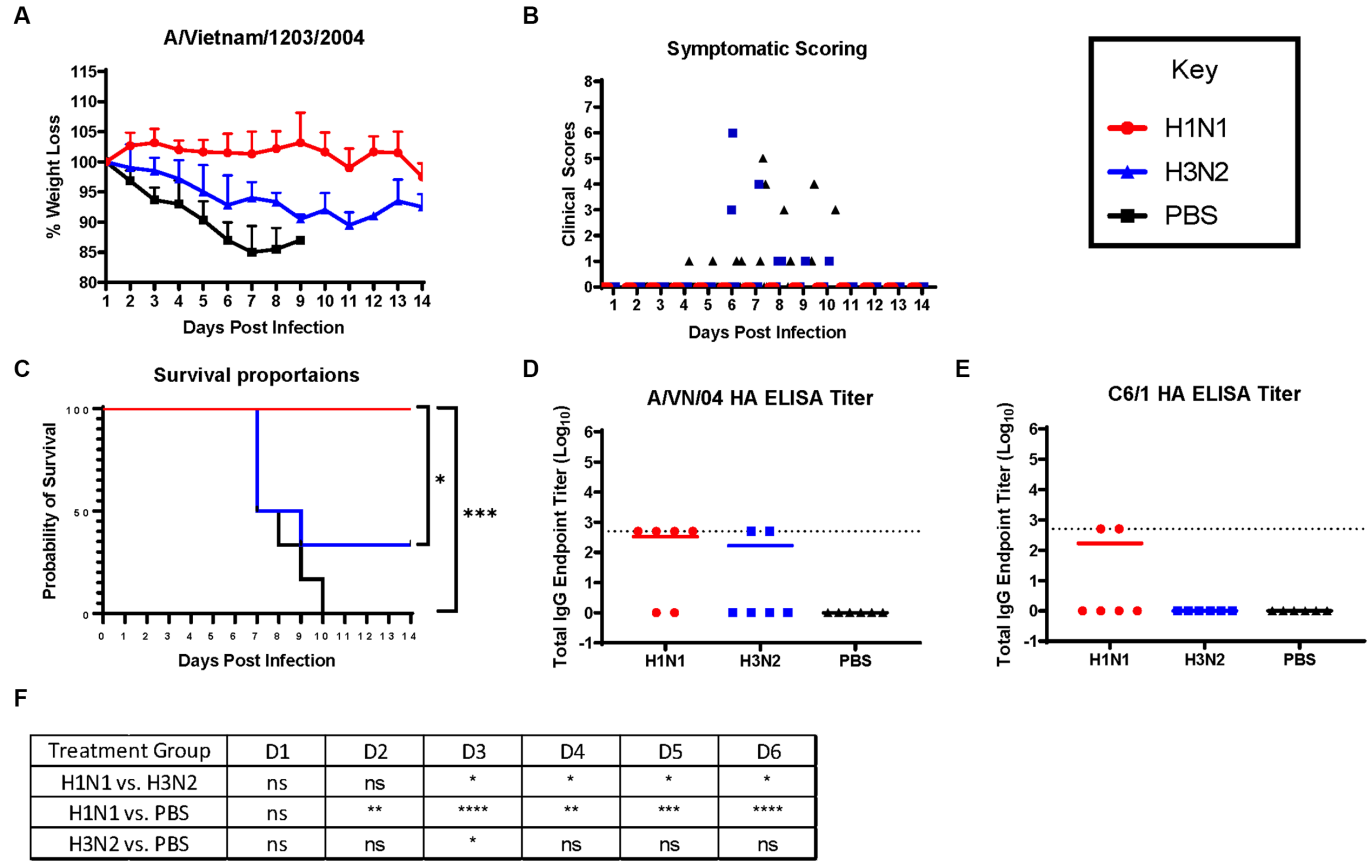
H1N1 imprinting provides complete protection against H5N1 challenge in naïve female ferrets. Survival was assessed in each pre-immune group and control. **(A)** Bodyweight curve of re-immunized female fitch ferrets. A/Sing/86 (H1N1), A/Pan/99 (H3N2), and control (PBS). **(B)** Clinical scores were recorded for each ferret following challenge. **(C)** Survival curves obtained following H5N1 HPAI challenge. Statistical analysis performed on PRSIM™ software using Log-rank Mantel-Cox test (H1N1 vs. PBS, *p* = 0.0004, H3N2 vs. PBS, *P* = ns, H1N1 vs. H3N2, *p* = 0.0178). **(D)** Detection of rHA VN/04 antibodies in pre-immune ferret sera collected on day 84 pre-infection. **(F)** Statistical analysis of body weight loss between imprinting groups post-exposure performed on PRSIM™ software using two-way ANOVA. Statistical differences were indicated as asterisks: *p < 0.05, **p < 0.01, ***p < 0.001, ****p < 0.001; ns, no significance. **(E)** Detection of rHA C6/1 antibodies in pre-immune ferret sera collected on day 84 pre-infection.

Ferrets were briefly anesthetized and intranasally challenged with the H5N1 influenza virus, VN/04 (1×10^5^ pfu). Weights and clinical scores were observed daily until ferrets reached a humane endpoint or until the end of the study (Figure 1). Ferrets that were previously inoculated with A/Sing/86 (H1N1) experienced little weight loss and had no clinical signs for 14 days following H5N1 viral infection (Figures 2A,B). However, ferrets that were imprinted with seasonal influenza virus A/Pan/99 (H3N2) experienced significant weight loss and developed multiple clinical symptoms as early as 6 days post H5N1 challenge (Figures 2A,B). Symptoms in this group including lethargy, weight loss, and neurological symptoms resulted in humane euthanasia (Figure 2C). H3N2 influenza virus-imprinted ferrets began to succumb to disease on day 6 post-challenge (Figure 2C), ultimately having a 66% lethality rate. Weight loss comparison between H1N1 and H3N2 influenza virus-imprinted ferrets was statistically significant between day 3 and day 6 post-challenge (Figure 2F). Ferrets that were inoculated with PBS in-lieu of seasonal influenza virus experienced significant weight loss (Figure 2A) and severe clinical symptoms (Figure 2B) and ultimately resulted in 100% mortality (Figure 2C) within 10 days following H5N1 influenza virus inoculation.

Sera collected from convalescent ferrets immediately prior to A/VN/04 viral challenge were tested for antibody reactivity against the HA protein of the A/VN/04 viral challenge strain in an ELISA binding assay (Figure 2D). Four out of six ferrets imprinted with the H1N1 virus had cross-reactive antibody titers against the HA of the H5N1 viral challenge strain A/VN/04 (Figure 2D), whereas only two ferrets imprinted with the H3N2 influenza virus had cross-reactive antibodies against the A/VN/04 HA protein. Interestingly, the two ferrets that had A/VN/04 HA cross-reactive antibodies survived challenge against the A/VN/04 H5N1 virus (Figures 2C,D). Additional ELISA assays confirmed seroconversion to the pre-immune challenge strains 14 days after inoculation (Supplementary Figure S1). To elucidate the mechanisms of antibody protection against H5N1 viral challenge, ferret sera were tested for binding antibodies against chimeric HA protein C6/1. Binding was detected against the specific HA chimeric protein with cross-reactive stalk antibodies. Only two ferrets from the H1N1 imprinted group had HAI titers against the C6/1 chimeric HA protein. From this preliminary data, we conclude that influenza virus group imprinting determines the ability to recall cellular immune responses and mount a protective response against pandemic H5N1 challenge that does not include an anti-stem binding antibody.

### 3.2 Sequential infection with a group 2 virus does not inhibit group 1 influenza virus imprinting

**Figure 3:**
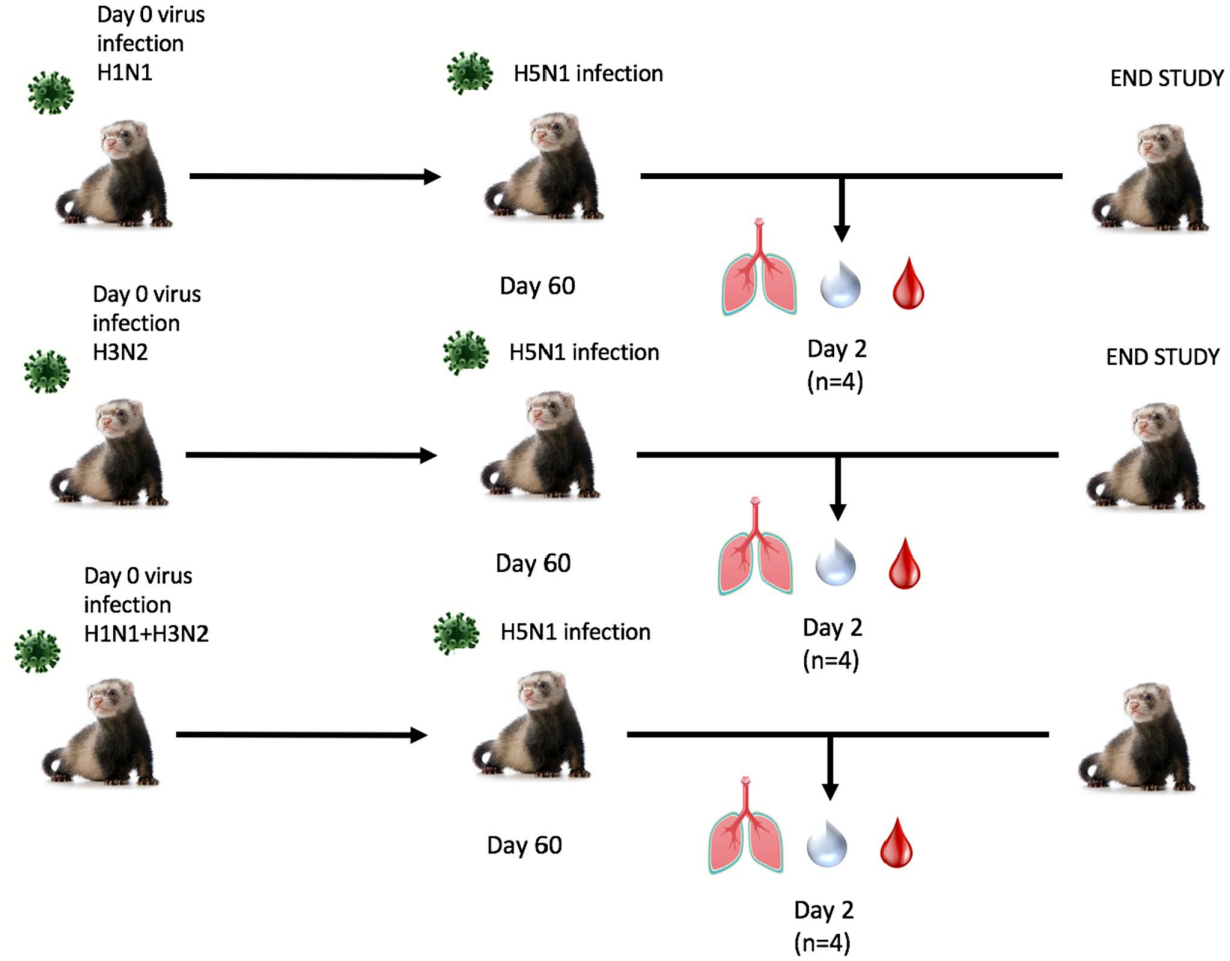
Schematic representation of secondary pre-immune ferret study. Male fitch ferrets (*n* = 12) were intranasally infected with live virus at 4–6 months of age using either H1N1 (A/Mar/43), H3N2 (A/HK/68), or both in a sequential infection. Sequentially infected ferrets were allowed to recover for 30 days between challenges. All groups recovered for 60 days and were challenged intranasally using highly pathogenic avian influenza (HPAI) H5N1 A/VN/04 (PFU 1×10^5^). Studies using HPAI were performed in a BSL3 select agent accredited facility. Ferrets were monitored for 14 days following challenge.

**Figure 4:**
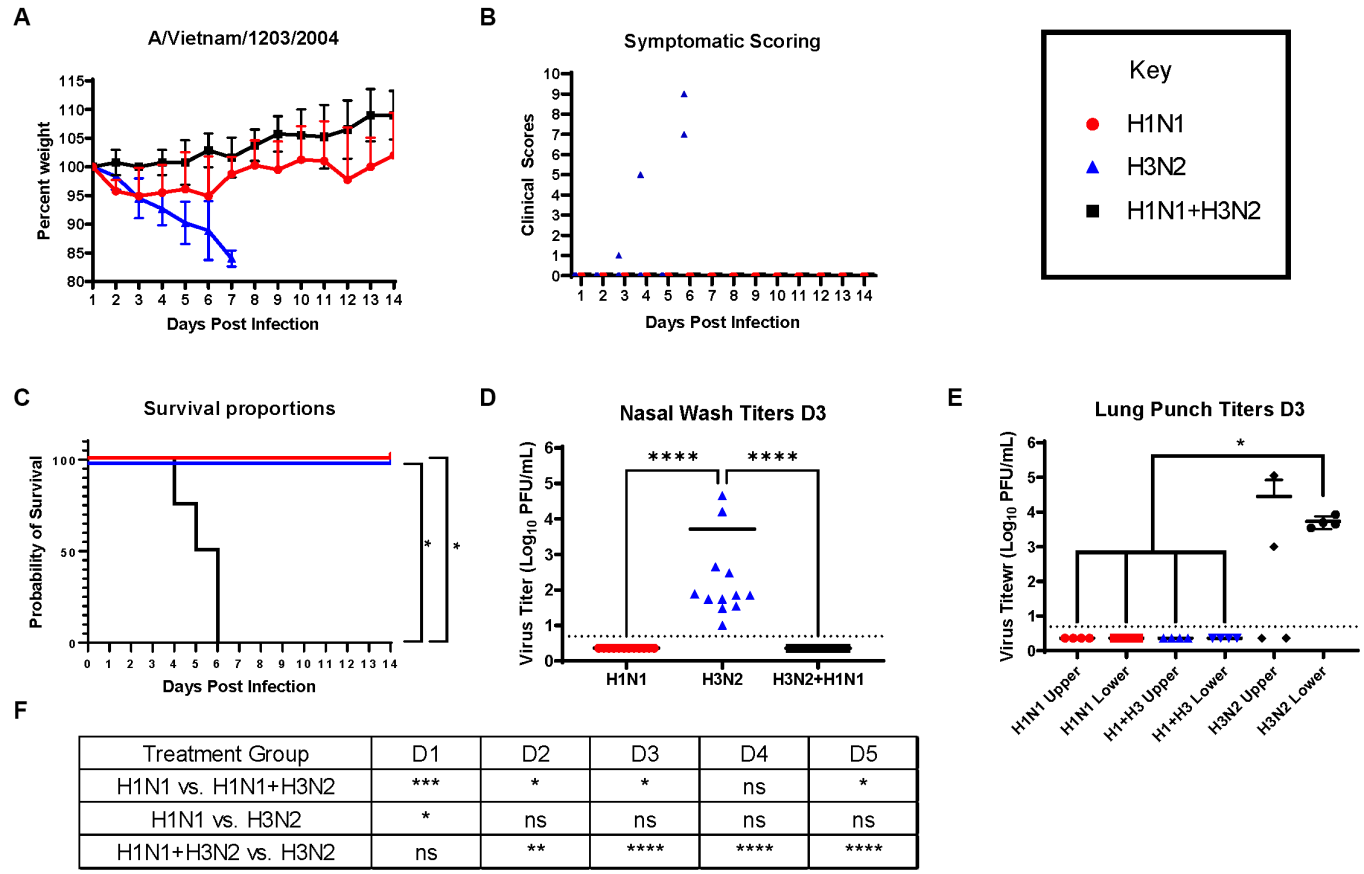
Sequential infection with a group 2 HA influenza virus does not deter primary group 1 imprinting protection. **(A)** Bodyweight curve of pre-immunized male fitch ferrets, *n* = 12 per group. **(B)** Clinical scores were recorded daily for each ferret following challenge. **(C)** Survival curves were obtained following H5N1 HPAI challenge. Statistical analysis performed on PRSIM™ using Log-rank Mantel-Cox test (H1N1 vs. H1N1 + H3N2, *p = 0.0091*, H1N1 vs. H3N2, *P* = ns, H3N2 vs. H1N1 + H3N2, *p = 0.0091*). **(D)** Nasal wash samples taken on Day 2 PI tested for detectable viral titers using plaque assay (plaque forming units = PFU). Statistical analysis was performed on PRISM™ using one-way ANOVA non-parametric Kruskal-Wallis test (*p < 0.0001*). Dunn’s multiple comparison test analysis was calculated and the results are indicated on the graph as asterisks: *p < 0.05, **p < 0.01, ***p < 0.001, ****p < 0.001; ns, no significance. **(E)** Lung punch samples taken on Day 2 PI tested for detectable viral titers using plaque assay (plaque-forming units = PFU). Statistical analysis was performed on PRISM™ using one-way ANOVA non-parametric Kruskal-Wallis test (*p < 0.0038*). Dunn’s multiple comparison test analysis was calculated and the results are indicated on the graph as asterisks: *p < 0.05, **p < 0.01, ***p < 0.001, ****p < 0.001; ns, no significance. **(F)** Statistical analysis of body weight loss between day 1 and day 5 post-infection (PI) performed on PRISM™ using two-way ANOVA. Statistical differences were calculated and indicated as asterisks: *p < 0.05, **p < 0.01, ***p < 0.001, ****p < 0.001; ns, no significance.

Next, ferrets were sequentially infected with Group 2 HA (H3N2) influenza viruses to determine whether the memory response of Group 1 HA (H1N1)-imprinted ferrets was skewed and deterred protection against pandemic H5N1 infection. This study also utilized distinct H1N1 and H3N2 influenza viral strains to further test the hypothesis of influenza viral Group 1 HA imprinting eliciting cross-reactive protection against Group 1 HPAI viral challenge and that protection is not based on specific H1N1 strains. Male naïve ferrets (*n* = 12) were inoculated with either A/Mar/43 viral strain (H1N1) or A/HK/68 viral strain (H3N2) or were sequentially infected first with A/Mar/43, then A/HK/68 30 days later. Ferrets in all groups were allowed to convalesce for 60 days following the last intranasal infection and were moved into a high-level ABSL-3 facility prior to challenge with H5N1 influenza virus, A/VN/04 (Figure 3). Similar to the previous study, ferrets that were imprinted with an H1N1 influenza virus had no weight loss (Figure 4A), no clinical symptoms (Figure 4B), and no mortality (Figure 4C) following H5N1 influenza viral challenge. Ferrets that were imprinted with an H3N2 influenza virus had severe and statistically significant weight loss (Figure 4A) and increased clinical scores (Figure 4B) and had 100% mortality (Figure 4C) within 7 days following H5N1 influenza viral challenge. H3N2 influenza virus-imprinted ferrets had severe signs of disease that reached humane endpoints prior to losing 20% of their original weight (Figures 4A,B). Most noticeably, H3N2 influenza virus-imprinted ferrets developed neurological symptoms, extreme lethargy, and hind limb paralysis prior to losing 75% of their original body weight (Figure 4B) and had a range of viral titers collected from nasal wash samples (Figure 4D). Similar to H1N1influenza virus-imprinted ferrets, H1N1 influenza virus infection followed by H3N2 influenza virus infection had little to no weight loss (Figure 4A) and no clinical symptoms (Figure 4B), and there was no mortality (Figure 4C). There was no statistical significance in ferret weights between H1N1 and H3N2 pre-immune groups on day 1 post-infection (Figure 4F). However, ferrets administered H1N1 followed by H3N2 influenza viruses and ferrets infected with H3N2 virus only had statistically lower weights on days 2–5 PI than ferrets infected with H1N1 influenza viruses only (Figure 4F). This analysis between weight curves suggests that sequentially inoculated ferrets experienced greater weight gain compared to the H1N1 pre-immunized group (Figure 4F). It is important to note here that the age of the male ferrets in this group, 4–6 months, influences weight curves as younger ferrets commonly gain weight throughout an experiment, whereas adult ferrets’ weight is stabilized. Therefore, the lack of weight gain in young ferrets can also be indicative of disease.

Nasal washes from all ferrets (*n* = 12) were collected on day 3 post-challenge to determine viral titers in the nasal cavity. As expected, only ferrets that were imprinted with the H3N2 virus were found to contain measurable virus in the nasal wash specimens (Figure 4D). In addition to nasal washes, ferrets from each group (*n* = 4) were humanely euthanized and lung punches were taken from the upper and lower quadrants of the right lungs. Plaque assays were performed to determine viral titers; upper and lower lung punches taken from ferrets pre-immunized with the H1N1 influenza virus or sequentially infected with the H1N1 and H3N2 influenza viruses had no viral lung titers (Figure 4E). However, punches taken from H3N2 influenza virus-infected ferrets had high titers in both upper and lower quadrants (Figure 4E).

Serological data taken from a subset of ferrets (*n* = 4) was tested for cross-reactive antibodies against A/VN/04-HA and C6/1-HA recombinant protein. H1N1 pre-immunized ferret sera contained detectable antibody titers against the A/VN/04-HA protein, whereas H3N2 pre-immune ferrets did not contain detectable antibody titers against A/VN/04 HA (Supplementary Figure S2A). Sera tested against the chimeric C6/1 stalk protein revealed no significant differences between pre-immune groups (Supplementary Figure S2B), further solidifying our hypothesis that HA-stalk antibodies do not aid in the protection against heterosubtypic H5N1 viral challenge.

### 3.3 Group 1 HA from H2N3 virus provides protection from heterosubtypic challenge of A/VN/04 H5N1 virus

**Figure 5:**
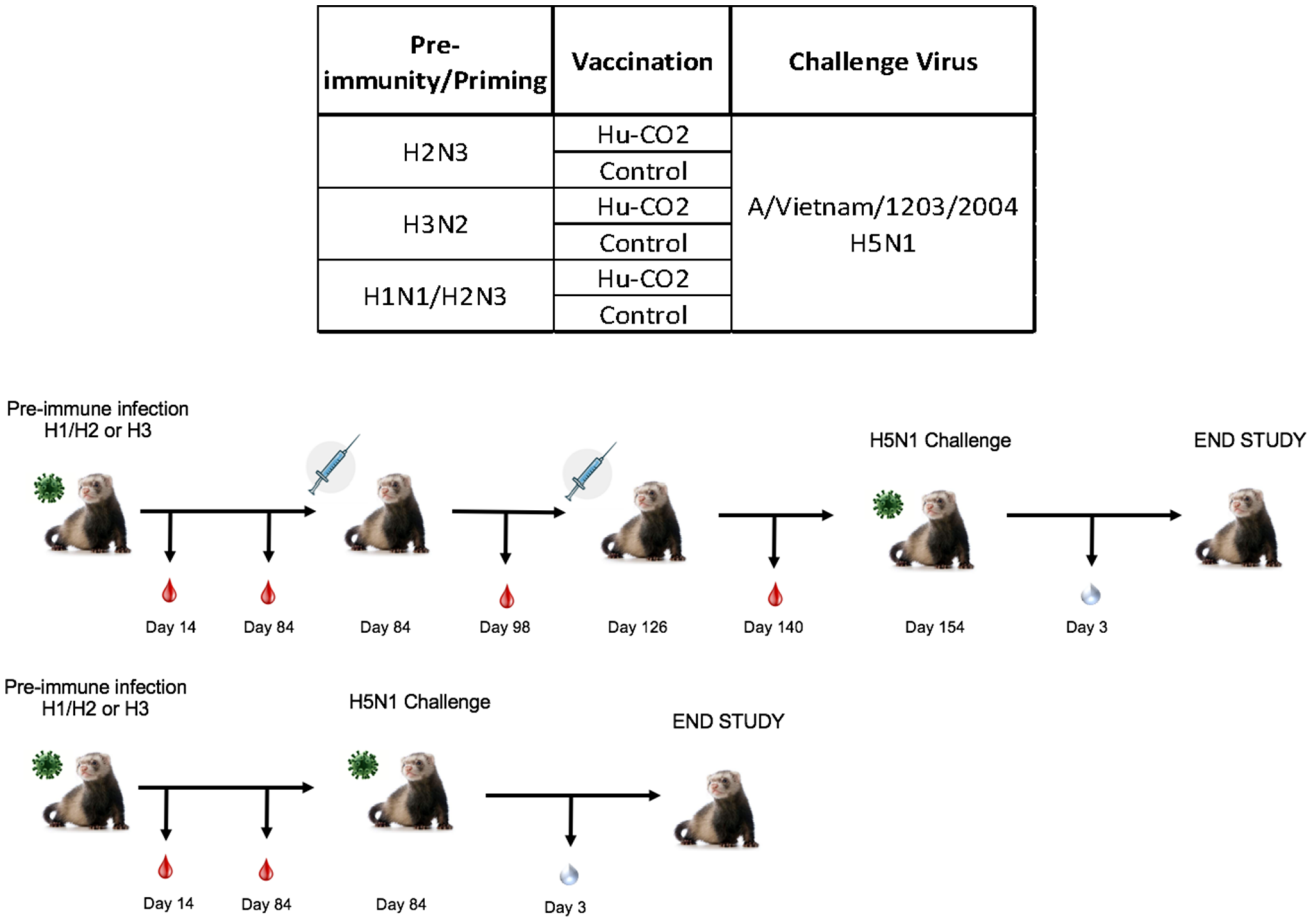
Schematic representation of tertiary pre-immune ferret study. Female fitch ferrets aged 9–12 months were intranasally infected with either an H3N2 virus (A/PC/73, *n* = 8), H2N3 (A/sw/MO/06), or a sequential infection of H1N1 + H2N3 (A/CA/09 followed by A/sw/M0/06). Ferrets were then intramuscularly vaccinated on a prime-boost regimen with either a Hu-CO 2 VLP formulated with Addavax™ adjuvant or PBS (control). Then, 28 days following vaccination, all groups were challenged intranasally using highly pathogenic avian influenza (HPAI) H5N1 A/VN/04 (PFU 1×10^5^). Studies using HPAI were performed in a BSL-3 select agent accredited facility. Ferrets were monitored for 9 days following challenge.

**Figure 6:**
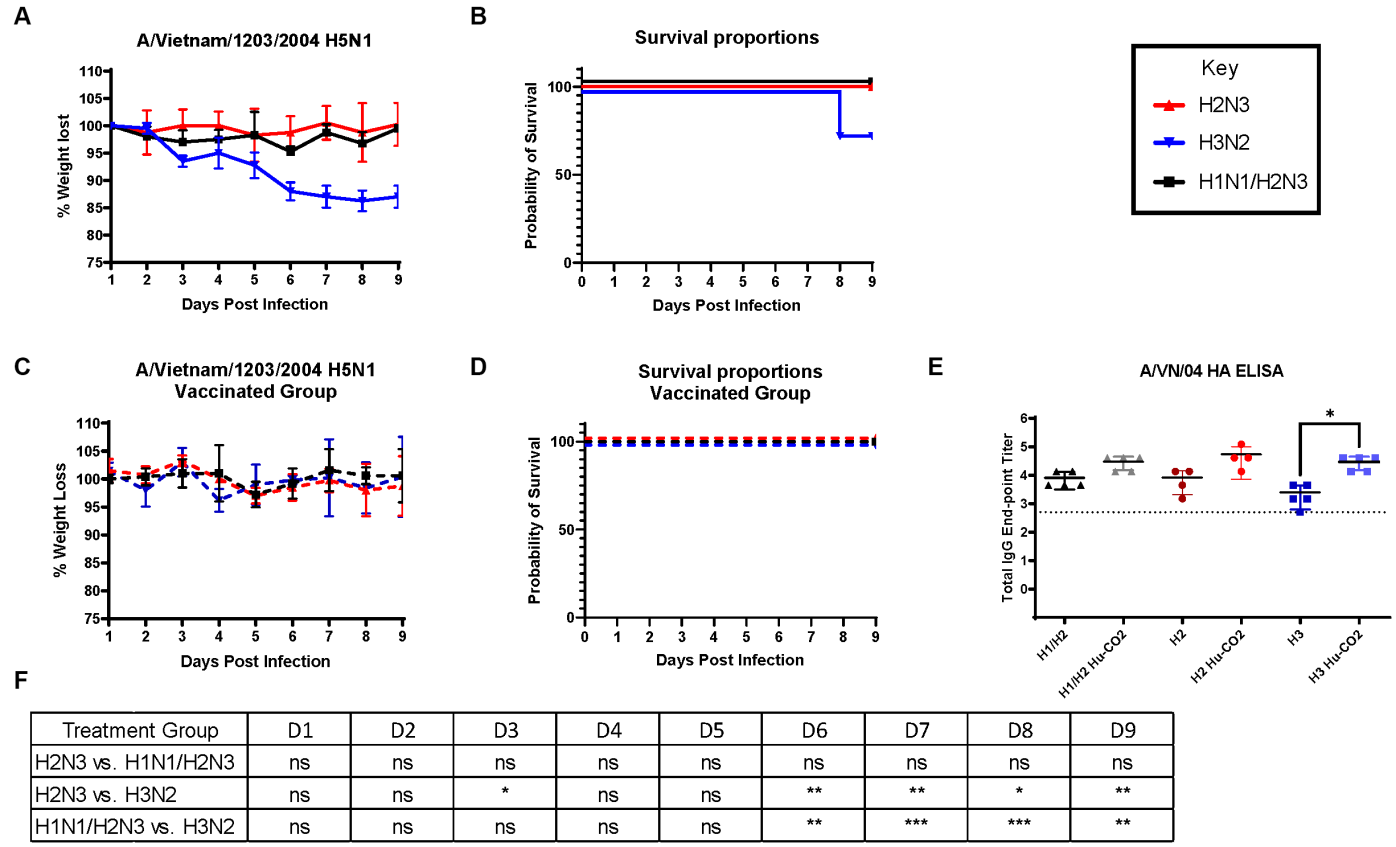
Influenza H2 imprinting protects ferrets from HPAI lethal challenge. **(A)** Bodyweight curve of pre-immunized female fitch ferrets, *n* = 4 per group. **(B)** Survival curves were obtained following H5N1 HPAI challenge. **(C)** Bodyweight curves of vaccinated pre-immunized female fitch ferrets, *n* = 4 per group. **(D)** Survival curves of vaccinated ferrets obtained following H5N1 HPAI challenge. **(E)** Statistical analysis of body weight loss between imprinting groups post-exposure performed on PRISM™ using two-way ANOVA. Statistical differences were calculated and indicated as asterisks: *p < 0.05, **p < 0.01, ***p < 0.001, ****p < 0.001; ns, no significance.

Although ferrets with pre-existing immunity to H1N1 influenza viruses are protected against H5N1 influenza virus challenge, further determination of group 1 HA viral inoculation elicits protective immune responses against H5N1 viral challenge. Female ferrets (6–8 months) were pre-immunized with either an H3N2 (A/PC/73, *n* = 3), H2N3 (sw/MO/06, *n* = 4), or sequential vaccination of H1N1 then H2N3 (A/CA/09 and A/sw/MO/06, *n* = 4) and were allowed to recover for 84 days prior to challenge with H5N1 virus (Figure 5). Aligning with previous data, pre-immune H3N2 viral ferrets experienced significant weight loss compared to H2N3 (group 1) pre-immunized ferrets (Figures 6A,E). H3N2 pre-immune ferrets displayed a 33% mortality rate following H5N1 viral challenge (Figure 6B). However, H2N3-imprinted ferrets experienced no significant weight loss and all survived challenge, along with H1N1 and H2N3 sequentially infected ferrets (Figure 6B). Statistical analysis of weight loss using two-way-ANOVA (*p* = 0.0007) and Tukey’s multiple comparisons showed significant weight differences between H3N2 and H2N3 and/or H1 + H2 pre-immune groups on Day 6-Day 9 post-infection (Figure 6F).

H3N2 influenza virus-infected ferrets were vaccinated with Hu-CO-2 virus-like particle vaccines formulated with Addavax™. Four weeks following the last boost, ferrets were intranasally challenged with the H5N1 virus. There were no observable clinical symptoms, weight loss (Figure 6C), or mortality (Figure 6D) following challenge. Sera taken from Ferrets had an increase in HAI activity against the A/VN/04 virus on days 84 and 140 following vaccination (Figure 6E).

### 3.4 Recombinant protein vaccination protects ferrets from lethal challenge with H5N1 virus

**Figure 7:**
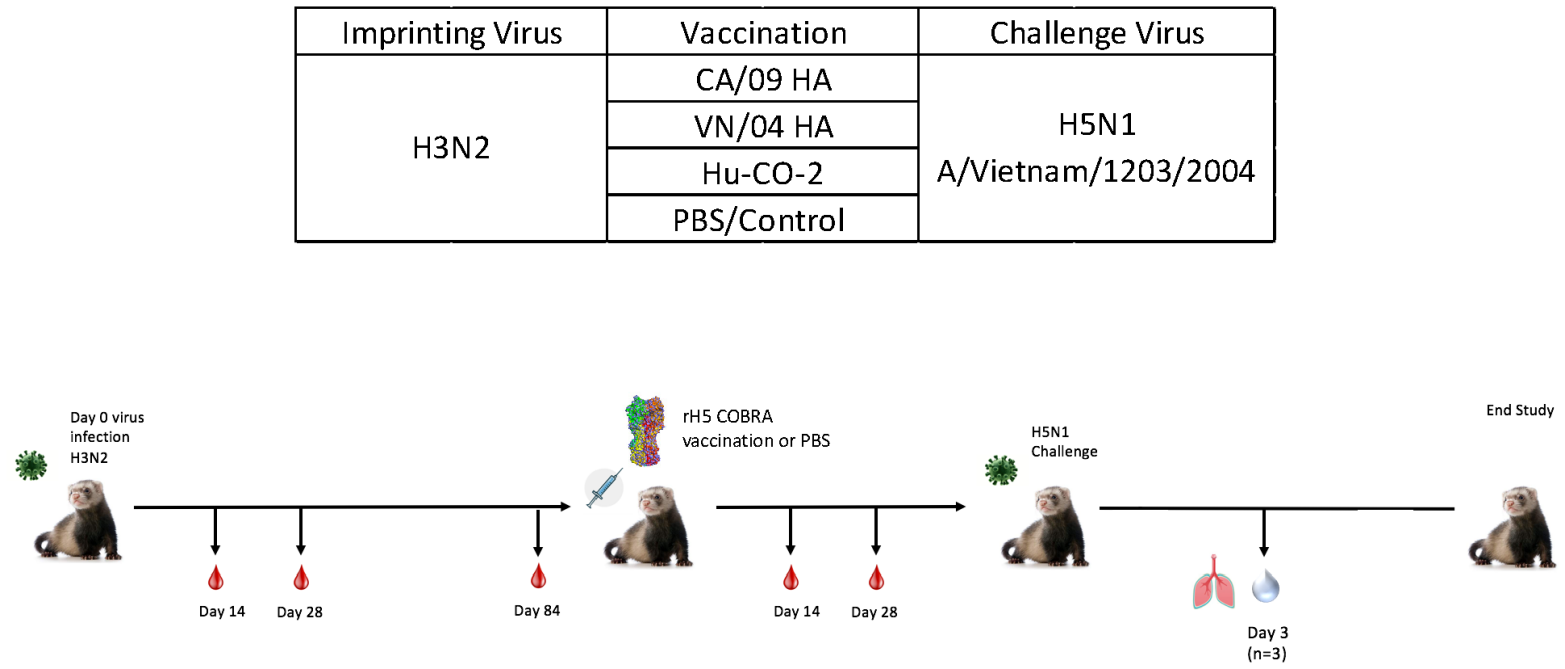
Schematic representation of H3N2 pre-immune vaccine study. Female fitch ferrets aged 9–12 months were intranasally infected using the H3N2 (A/TX/12) virus for pre-immune imprinting. After 84 days, ferrets were intramuscularly vaccinated with rHA formulated with Addavax™. Vaccines contained either A/CA/09-HA, A/VN/-4-HA, Hu-CO 2-HA, or PBS (control). Then, 28 days following vaccination, all groups were challenged intranasally using highly pathogenic avian influenza (HPAI) H5N1 A/VN/04 (PFU 1×10^5^). Studies using HPAI were performed in a BSL-3 select agent accredited facility. Ferrets were monitored for 14 days following challenge. **(E)** Ferret serum collected on day 84 and day 140 was tested for total IgG reactivity against A/VN/04 HA antigen in an ELISA assay. Limit of detection for this assay is represented by dotted line. F) (previously E).

While ferrets imprinted with group 1 influenza viruses can induce protective immune responses against H5N1 challenge, the exact mechanism of protection is unclear. To better understand this phenomenon, both female and male ferrets were pre-immunized with an H3N2 influenza virus (A/TX/12) and then vaccinated with HA or NA protein vaccines followed by challenge with an H5N1 A/VN/04 influenza virus (1×10^5^ pfu/ml) (Figure 7).

**Figure 8:**
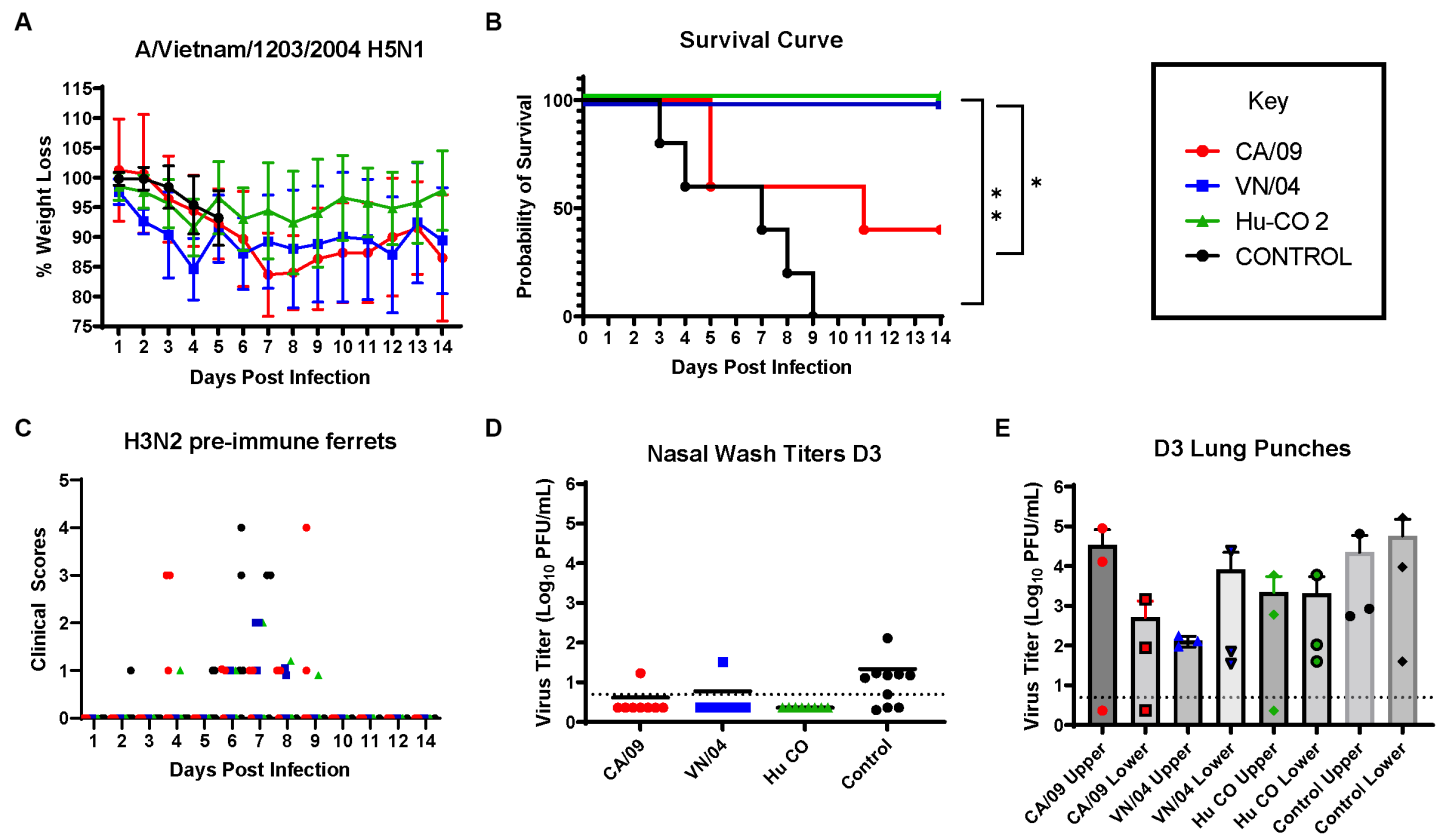
H3N2 pre-immune ferrets vaccinated with Human-COBRA 2 HA are rescued from lethal challenge with HPAI virus. **(A)** Bodyweight curve of pre-immunized control (*n* = 10) and vaccinated female fitch ferrets, *n* = 8 per vaccine group. **(B)** Survival curves obtained following H5N1 HPAI challenge. Statistical analysis performed on PRSIM™ software using Log-rank Mantel-Cox test (Hu-Co 2 or A/VN/04 vs. A/CA/09, *p =* 0.0486, Hu-Co2 or A/VN/04 vs. PBS, *p* = 0.0018). **(C)** Clinical Scores were recorded for each ferret following H5N1 challenge. **(D)** Nasal wash samples obtained from anesthetized ferrets on Day 3 PI were tested for detectable viral titers using plaque assay (PFU, plaque forming units). **(E)** Lung-punch samples taken on Day 2 PI were tested for detectable viral titers using plaque assay (PFU, plaque forming units).

Pre-immune ferrets vaccinated with A/CA/09-HA rapidly lost weight and by day 5 post-infection, 50% of the ferrets had died, which was similar to mock vaccinated ferrets except that all the ferrets died between days 3 and 9 post-infection (Figures 8A,B). Ferrets vaccinated with either HA from A/VN/04 or Hu-CO 2 all survived challenge (Figure 8B). However, ferrets vaccinated with Hu-CO-2 rHA had less than 5% weight loss, whereas ferrets vaccinated with A/VN/04 rHA lost between 10 and 15% of their original body weight. Ferrets pre-immune to H3N2 influenza viruses that were mock vaccinated had neurological signs or were found to be moribund before they reached the 75% weight cut-off and experienced high clinical scores (Figure 8C) with high viral nasal wash titers in control group ferrets (Figure 8D), whereas vaccinated ferrets had no significant viral nasal wash titers (Figure 8D).

On day 3 post-challenge, lung punches were collected from the upper and lower quadrants of each lung. All ferrets had detectable influenza virus in both upper and lower lung quadrants. Control and A/CA/09-HA-vaccinated ferrets had the highest upper quadrant viral lung titers (Figure 8E). Ferrets vaccinated with A/VN/04-HA had lower viral titers in the upper respiratory tract compared to Control and A/CA/09-HA-vaccinated ferrets.

Ferrets seroconverted to the pre-immune strain A/TX/12 (Supplementary Figure S3), as well as all the vaccines used to vaccinate each ferret (Supplementary Figure S3D). Sera collected from ferrets with A/VN/04-HA protein had detectable IgG anti-A/CA/09-HA antibodies and A/CA/09-HA-vaccinated ferrets had antibodies against A/VN/04-HA; however, these antibodies were not protective in challenge (Supplementary Figure S3B). Histological samples taken 3 days following infection showed that ferrets vaccinated with the Hu-CO 2 vaccine had lower cellular infiltrates when compared to A/CA/09- and A/VN/04-vaccinated ferrets (Supplementary Figure S4), and all three vaccine groups improved lung pathology in comparison to unvaccinated H3N2 primed controls (Supplementary Figure S4D).

## 4 Discussion

Overall, there are patterns of influenza group imprinting that affect the clinical outcomes of H5N1 virus infections in the ferret model of disease. This study found that group 1 HA influenza A viruses (IAV) elicited heterosubtypic protection against lethal challenge of highly pathogenic avian influenza virus (HPAIV) H5N1 (Figures 2C, 4C, 6B). This data correlates with multiple studies predicting the heterosubtypic immune response elicited by group 1 viruses against group 1 pandemic strains. Specifically, heterosubtypic immunity has been well established in mouse and ferret models of disease (33–37). In lethal challenge studies, naïve female BALB/c mice intranasally inoculated with PR8 (H1N1) virus and subsequently challenged with X31 (H3N2 virus) experienced accelerated viral clearance in the lungs 5 days after challenge (34). However, PR8-priming did not offer complete protection from X31-induced disease in these mice (34). A similar study performed in mice evaluated the heterologous protection of a recombinant influenza virus containing internal influenza B genes and neuraminidase and HA from H1 or H3 virus (35). Mice inoculated with the recombinant B-H1 virus exhibited enhanced protection against H5N1 viral challenge, whereas mice primed with the B-H3 virus or influenza B virus displayed higher morbidity and lower survival rates (35).

The hypothesis of influenza group-dependent imprinting and immunity is further established using Ferret disease models, as they are considered the gold standard for influenza virus infection. In this study, ferrets that received the H3N2 A/Sing/86 virus were afforded partial protection against mortality, whereas male ferrets inoculated with H3N2 strain A/HK/68 all succumbed to disease within 6 days following challenge. It is important to note here that although the dosage of the virus was slightly increased to compensate for the increased mass of the male ferret, the same viral strain and passage were used. In hindsight, a similar study should be performed in female ferrets inoculated with the A/HK/68 strain to correlate mortality and sex differences. This, however, does not lead us to believe that the H3N2 virus strain A/HK/68 would elicit significant protective heterosubtypic immunity to the extent of group 1 HA imprinting. Previous studies involving H3N2 virus pre-immunity also display varying results with different H3N2 strains (36, 37). Ferrets inoculated with A/Victoria/361/2011 H3N2 virus and subsequently challenged with the H5N1 virus all succumbed to systemic disease and encephalitis (37). However, ferrets made pre-immune with H3N2 strain A/Perth/16/2009 all survived challenge and experienced lower viral replication and weight loss and fewer clinical symptoms compared to PBS control (36).

The protective effects of group 1 HA imprinting did not falter, however, differing with the viral H1N1 strains both in this study and previous studies (36, 37). Male ferrets primed with H1N1 strain A/Mar/43 showed complete protection from clinical symptoms following viral challenge. This was also true for group 1 HA strain A/sw/MO/06 (H2N3)-primed ferrets, where lethal challenge with the H5N1 virus resulted in no weight loss or clinical symptoms. The investigation of H2 pre-immunity is especially relevant in the context of vaccine preparedness for the elderly population. The influenza virus strain H2N2 circulated from 1957 to 1968, resulting in the Asian Flu pandemic in East Asia (33). During this pandemic, an estimated 1.1 million deaths occurred worldwide (33). Individuals born during this time have also shown decreased severe clinical outcomes against H5N1 virus infection (14). Utilizing this strain not only strengthens influenza group-dependent immunity but also removes the possible effects of matching neuraminidase-induced antibodies against avian-derived N1.

Although group 1 HA-primed ferrets were protected from H5N1 virus-induced disease, the exact mechanism of protection is not well understood. We first thought to investigate the generation of neutralizing HA stem antibodies following pre-immunity as a protective mechanism against disease. Influenza HA-stem antibodies are commonly studied due to their therapeutic potential against IAV disease and are often targeted for vaccine design due to their conserved epitopes (33, 38–41). The chimeric HA protein containing an H6 head and H1 stalk was utilized in this study to detect HA stalk binding IgG in pre-immune ferret sera, assuming the sera would not contain cross-reactive Abs binding the H6 HA head. Although there were many ferrets that contained cross-reactive stem IgG, there was no correlation between HA-stalk Ab and reduced clinical symptoms (Figure 2E and Supplementary Figure S3C). Previous mouse studies have found a positive correlation with stalk-reactive IgG levels and heterologous virus protection (42). However, the lack of cross-reactive antibodies has been documented in pre-immune ferrets models of disease (34).

In the absence of cross-reactive antibodies, protection against heterologous strains of influenza can be attributed to cellular immunity. T-cell-mediated memory responses against heterologous viral challenge have been studied in ferret models of disease (43, 44). Naïve ferrets imprinted with H1N1 IAV experienced decreased viral shedding and clinical symptoms following H3N2 viral infection but were not entirely protected from disease (43). Ferrets inoculated with H1N1 influenza viruses lacked serum antibodies against the H3N2 virus but had a peak in interferon gamma-secreting T cells 11 days following H3N2 viral challenge (43). In ferrets receiving live attenuated influenza vaccine (LAIV) in human studies, live attenuated influenza vaccination (LAIV) in young children elicits both serological and cellular immunity (45). LAIV mimics influenza viral infection and induces robust CD4+ and CD8+ T-cell activation, which cannot be achieved through TIV (45). Testing serum IgG antibodies in pre-immune ferrets provides a small insight into the mechanism of protection of group 1 imprinted ferrets against H5 challenge. Cellular or humoral responses against neuraminidase or matrix proteins were not studied here. However, it is clear that Group 1 H2-imprinted ferrets lacking a matching neuraminidase (N3) offered complete protection from H5N1 virus morbidity and mortality.

In attempts to remedy the lack of complete protection from H3N2 viral inoculation, ferrets were intramuscularly vaccinated with monovalent wildtype rHA or with the Human COBRA 2 vaccine. Vaccinated ferrets did not experience clinical symptoms or mortality following challenge. This was also true for the rHA A/VN/04-vaccinated ferrets. This may lead to the conclusion that monovalent strains of vaccination are equally efficacious to chimeric-COBRA rHA. This, however, can be disproven by decades-long research showing the broad reactivity of Hu-CO2 against variant strains of H5 in multiple animal models of disease (32, 46–50). From here, we can conclude that the H3N2 group 2 imprinting can be rescued by pandemic vaccination of a chimeric H5 HA protein expressing Hu-CO 2 or through vaccination with a matching monovalent strain. It is also important to note that the monovalent rHA vaccines were tested using a one-shot regimen in order to simulate dose sparing in pandemic vaccination. Interestingly, 60% of A/CA/09 HA-vaccinated ferrets died and had clinical symptoms similar to H3N2 mock-vaccinated ferrets (Figures 8B,C). This could be due to the low immunogenicity of the A/CA/09 HA protein; however, IgG titers from A/CA/09 HA-vaccinated ferrets had similar IgG titers to A/CA/09 HA and Hu-Co-2 rHA-vaccinated ferrets, which all survived H5N1 influenza virus challenge (Supplementary Figure S3). This further reiterates the lack of correlation between cross-reactive Abs to the challenge strain and protection.

These immune imprinting effects play a role in the development of a universal influenza vaccine. Many studies have led to the speculation that an individual’s birth year and subsequent viral imprinting determine the trajectory of future antibody responses to vaccination (12, 14, 33, 34). Therefore, vaccine studies performed in animal models lacking pre-immunity greatly delay the progression of designing a universal influenza virus vaccine. Vaccine studies in naïve ferret models have resulted in narrowly binding Ab repertoires, lacking the breadth and strength of responses in previously infected/pre-immune ferrets (51, 52). Vaccination in a naïve immunological background may also result in increased clinical symptoms following pandemic strain H1N1 A/CA/09 viral challenge, suggesting that vaccination in the absence of primary infection leads to the development of non-neutralizing antibodies (35).

The current H5N1 viral outbreak continues to devastate animal populations including poultry, wild birds, and wild mammals. The individuals responsible for maintaining poultry populations for food and survival are at imminent risk of contracting disease. In addition, the increased incident rate of H5N1 spillover into the human population increases the probability of viral mutation and spread, as has been seen in reports in Ecuador, Cambodia, and Chile (53–55). Vaccine design and implementation should be a top priority for pandemic preparedness, and the development of an immunogenic universal H5 vaccine will have to consider the pre-immune status of the individuals in question. The specific aims of universal influenza vaccine coverage include the following: 1. all influenza A and B viruses independent of the NA subtype; 2. historical and future influenza strains; 3. pandemic influenza strains; and 4. protection from zoonotic spillover (56, 57). These goals can be achieved by implementing a chimeric HA vaccine that targets conserved epitopes shared throughout H5 viral clades, addressing the problematic accelerated evolution of the H5 virus HA protein. In this study, we demonstrated the protective effects of group 1 influenza pre-immunity against H5N1 viral challenge, and the variable effects of group 2 H3N2 pre-immunity that can be rescued with a one-dose vaccination using a highly immunogenic broadly reactive HA antigen Human COBRA 2 formulated with adjuvant. The efficacy of the H5 Human COBRA 2 vaccine and its predecessors will continue to be investigated in animal models of disease (46), and further studies will be conducted to access immunogenicity in a pre-immunological background against new variant H5 viral strains.

## Data availability statement

The raw data supporting the conclusions of this article will be made available by the authors, without undue reservation.

## Ethics statement

The animal study was approved by Leanne Alworth-University of Georgia IACUC Office of Research Director. The study was conducted in accordance with the local legislation and institutional requirements.

## Author contributions

IN: Conceptualization, Data curation, Formal analysis, Investigation, Methodology, Writing – original draft, Writing – review & editing. HJ: Investigation, Methodology, Project administration, Supervision, Writing – review & editing. YH: Methodology, Validation, Writing – review & editing. AK: Conceptualization, Methodology, Project administration, Resources, Writing – review & editing. TR: Conceptualization, Funding acquisition, Project administration, Resources, Supervision, Visualization, Writing – review & editing.
