# Supplementary material for: Influenza virus immune imprinting dictates the clinical outcomes in ferrets challenged with highly pathogenic avian influenza virus H5N1
Source: Front Vet Sci. 2023 Dec 19;10:1286758. doi: 10.3389/fvets.2023.1286758 (PMC10759238; doi:10.3389/fvets.2023.1286758)
Supplement: Supplementary file 1 [file Data_Sheet_1.docx]

##
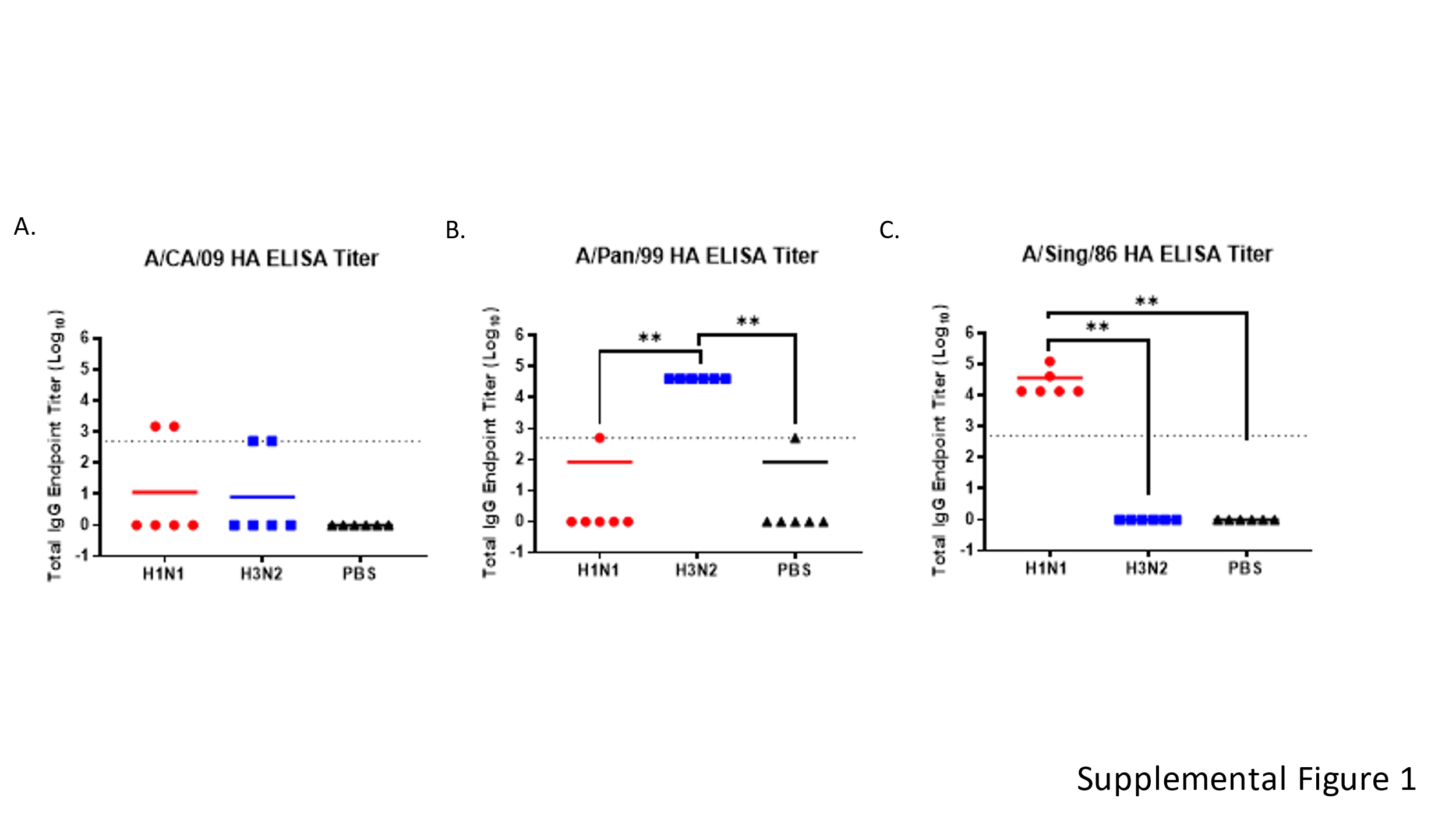
Supplementary Figures

## Supplementary Figure 1. Establishing seroconversion of female fitch ferrets intranasally inoculated with A/Sing/86, A/Pan/99, or PBS control. Sera from pre-immune imprinted ferrets was collected 84 days following intranasal inoculation of seasonal influenza virus. Diluted sera were tested for HA specific antibody reactivity against (A) A/CA/09, (B) A/Pan/99, and (C) A/Sing/86 HA soluble recombinant proteins in an ELISA assay. Statistical analysis was performed in PRISM™ using One-Way ANOVA non-parametric Kruskal-Wallis test (*P=0.002*) and Dunn’s Multiple comparisons test indicated as asterisks on graph: **P*<0.05, ** *P*<0.01, *** *P*<0.001, *****P*<0.001, ns= no significance.


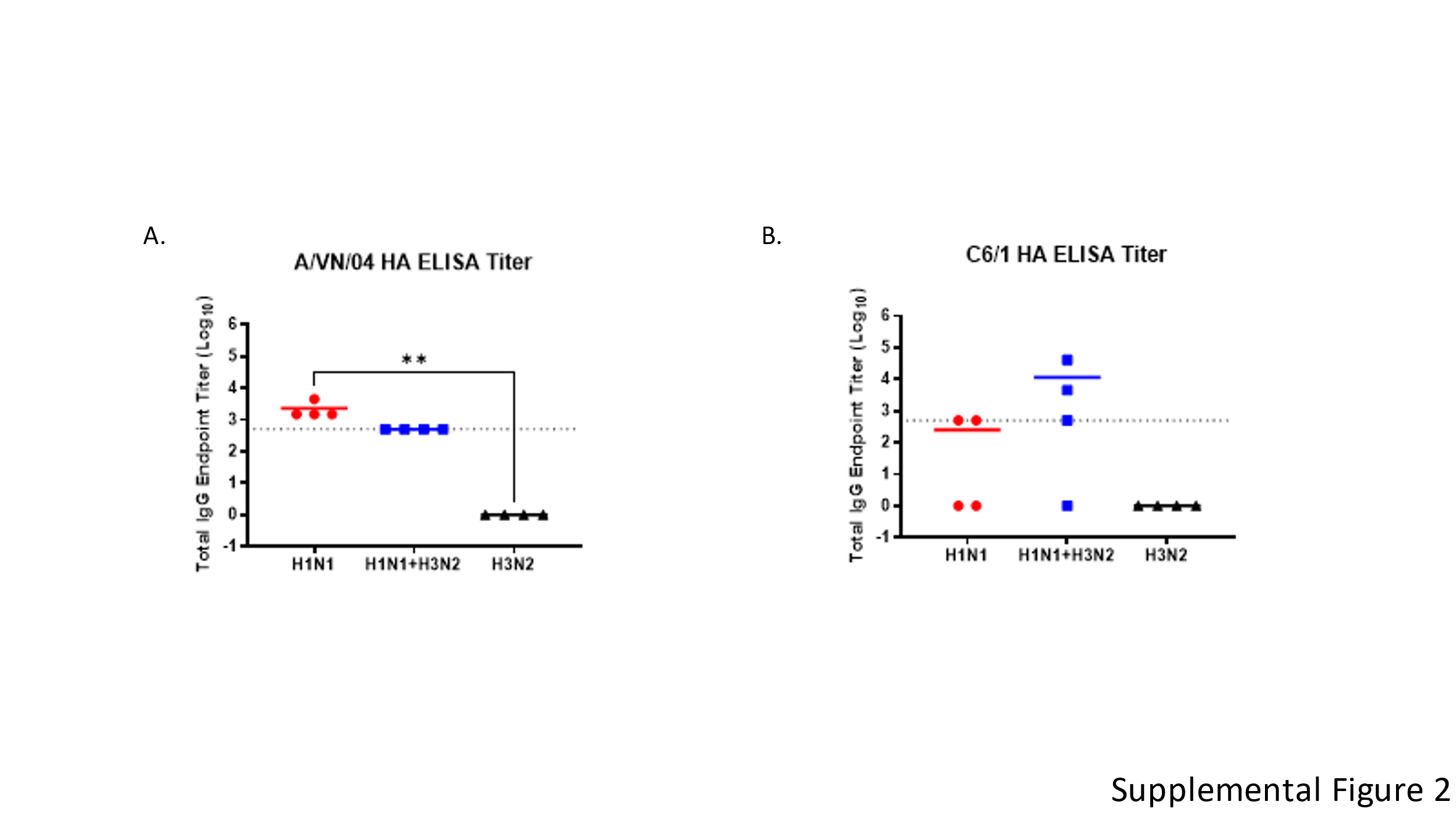


**Supplementary Figure 2: Hemagglutinin Stem directed antibodies are not indicative of heterosubtypic protection.** Sera from pre-immune imprinted ferrets was collected 60 days following intranasal inoculation of seasonal influenza virus. Diluted sera were tested for HA specific antibody reactivity against challenge strain. (A) A/VN/04, *P=0.002* and (B) C6/1, *P=ns* HA soluble recombinant protein in an ELISA assay. Statistical analysis was performed in PRISM™ using One-Way ANOVA non-parametric Kruskal-Wallis test and Dunn’s Multiple comparisons test indicated as asterisks on graph: **P*<0.05, ** *P*<0.01, *** *P*<0.001, *****P*<0.001, ns=no significance.


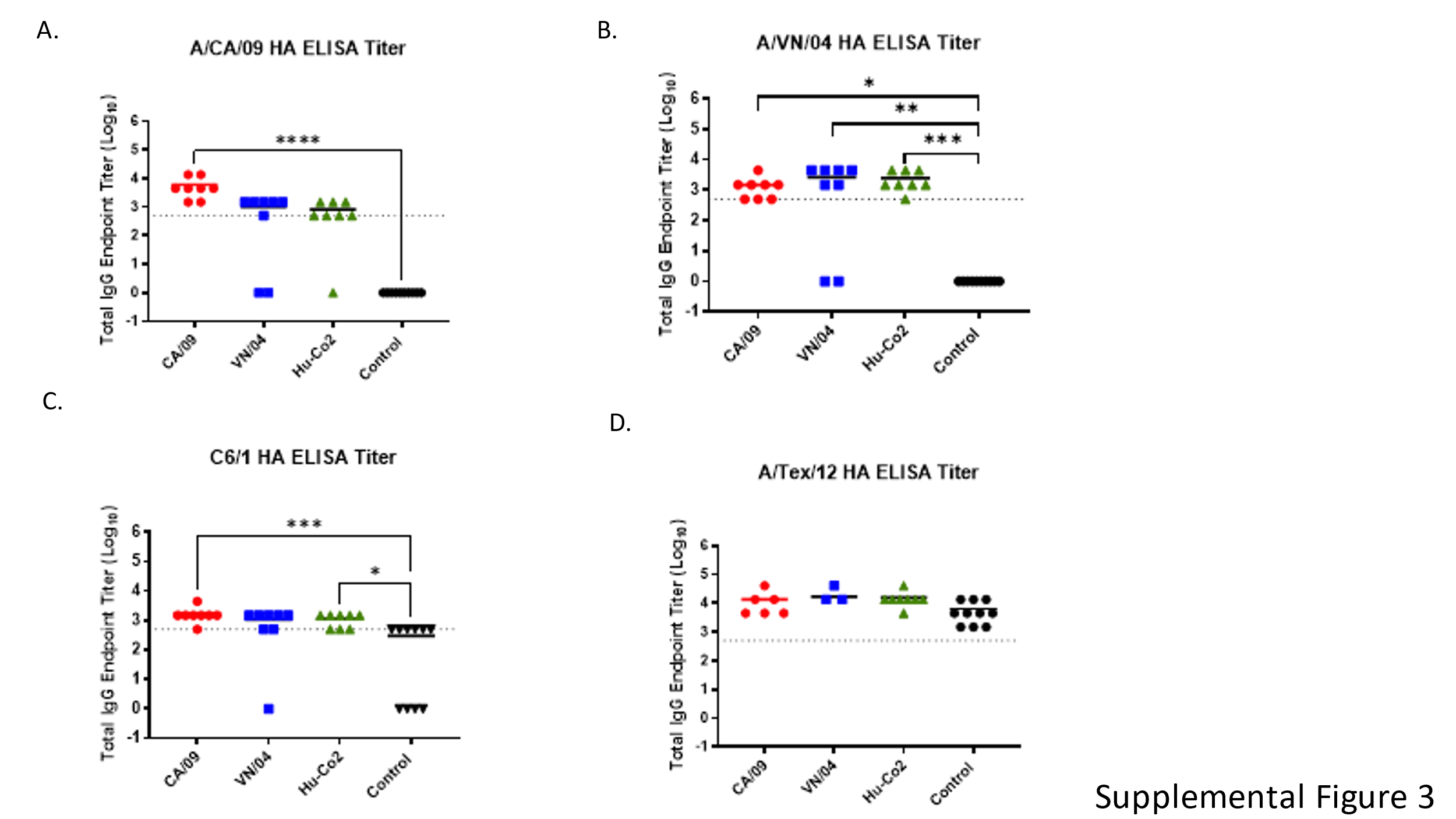


**Supplementary Figure 3: Vaccination with HU-Co2 soluble rHA elicits antibodies against A/VN/04 HA.** Sera from pre-immune imprinted ferrets was collected 28 days following vaccination with rHA. Diluted sera were tested for HA specific antibody reactivity against challenge strain (A) A/CA/09, *P=0.0001*, (B) A/VN/04, *P=0.0002* (B) C6/1, *P=0.0009*, and (D) A/Tex/12, *P=NS* HA soluble recombinant protein in an ELISA assay. Statistical analysis was performed in PRISM™ using One-Way ANOVA non-parametric Kruskal-Wallis test (listed above) and Dunn’s Multiple comparisons test indicated as asterisks on graph: **P*<0.05, ** *P*<0.01, *** *P*<0.001, *****P*<0.001, ns=no significance.


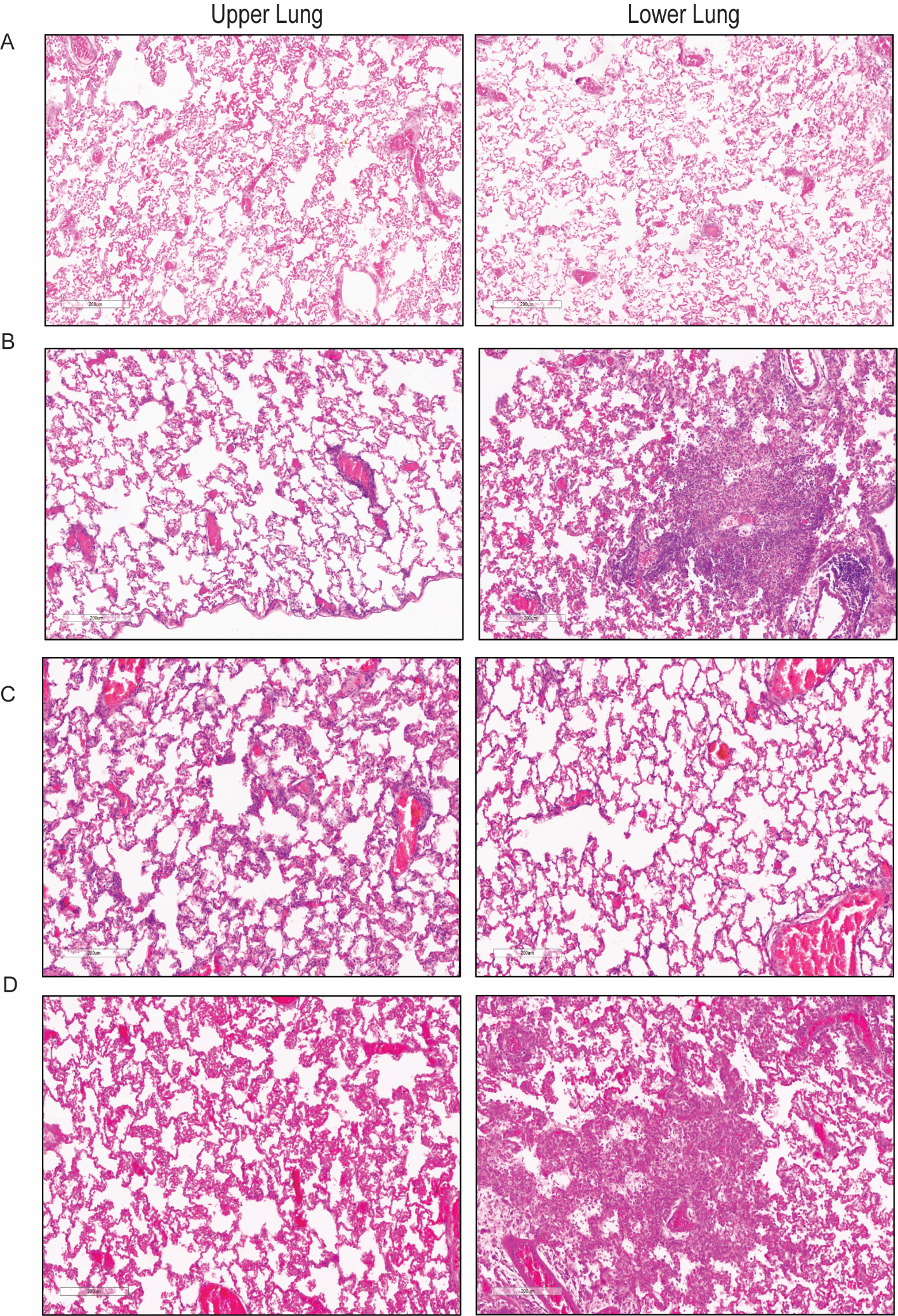


**Supplementary Figure 4**: **Lungs taken from ferrets on D3 post-challenge revealed Hu-CO2 HA vaccinated ferrets had less inflammation and cellular infiltrates than A/VN/04 or CA/09 vaccinated ferrets.** Samples were taken from each sample of upper and lower lungs. A) A/CA/09 HA vaccinated ferrets B) A/VN/04 HA vaccination C) Human CO2 vaccination and D) Control Vaccinated ferret lungs.
